# Capability, opportunity, and motivation: an across contexts empirical examination of the COM-B model

**DOI:** 10.1186/s12889-021-11019-w

**Published:** 2021-05-29

**Authors:** Taylor Jade Willmott, Bo Pang, Sharyn Rundle-Thiele

**Affiliations:** Social Marketing @ Griffith, Griffith University, 170 Kessels Road, Nathan, QLD 4111 Australia

**Keywords:** Diet, Eating, Intervention, Model, Nutrition, Overweight, Obesity, Physical activity, Theory, Weight gain

## Abstract

**Background:**

There is limited evidence for successful weight gain prevention interventions targeting young adults. Developing effective interventions necessitates a theoretical model that can identify barriers and enablers for healthy eating and physical activity among young adults to support weight management. This study empirically examines the utility of the COM-B model as a framework for intervention planning across two behavioural contexts: eating and physical activity.

**Methods:**

A cross-sectional survey research design was employed to empirically test the COM-B model in the contexts of young adult’s eating and physical activity behaviours. Informed by the Theoretical Domains Framework, pre-validated measures appropriate for capturing the latency of the COM (Capability, Opportunity, and Motivation) constructs were sourced. Both surveys (eating and physical activity) were administered online to two independent samples of young adults aged 18–35 years. Models were specified and tested using structural equation modelling.

**Results:**

A total of 582 (mean age = 22.8 years; 80.3% female) and 455 (mean age = 24.9 years; 80.8% female) participants were included in the physical activity and eating analyses, respectively. The COM-B model explained 31% of variance in physical activity behaviour and 23% of variance in eating behaviour. In the physical activity model (*N* = 582), capability and opportunity were found to be associated with behaviour through the mediating effect of motivation. In the eating model (*N* = 455), capability was found to be associated with behaviour through the mediating effect of motivation. Capability was also found to mediate the association between opportunity and motivation. Consistencies and variations were observed across both models in terms of COM indicators.

**Conclusions:**

Findings support the COM-B model’s explanatory potential in the context of young adult’s physical activity and eating behaviours. Barriers and enablers underlying young adult’s physical activity and eating behaviours were identified that represent potential targets for future intervention design. Further research is needed to validate present study findings across different populations and settings.

**Supplementary Information:**

The online version contains supplementary material available at 10.1186/s12889-021-11019-w.

## Background

The most rapid weight gain in the life course has been observed during the early twenties to mid-thirties [1, 2], with incident obesity at a younger age associated with increased risk of chronic disease and mortality in later adult life [2–4]. The causes of age-related weight gain are complex, encompassing a range of individual, social, and environmental factors that are often interrelated and interdependent. Young adulthood, defined by the age range of 18–35 years [5], is a transitional life stage in which young people encounter significant life changes, experience increasing independence, and adopt lasting behavioural patterns [6]. Adoption of unhealthful behaviours, including marked declines in physical activity [7–9], increases in sedentary behaviour [10, 11], and poor dietary habits [8, 12–16], during young adulthood has been associated with weight gain over time [17–19]. A longitudinal cohort study of 640 adolescents observed a 24% decrease in physical activity over a 12-year transition from adolescence to early adulthood [9]. Similarly, a cohort study of 773 young adults found 61.4% of young adult’s waking wear time to be spent sedentary [11]. A global analysis from 187 countries found young adults have the lowest diet quality compared with any other age group [16]. The adoption of unhealthful behavioural patterns during young adulthood are attributed to the significant life changes that occur during this transitional period such as moving out of the family home, relocating to new environments, beginning full-time work or further study, and establishing financial, residential, and employment stability [6]. In particular, the transition to higher education has been linked to weight gain and undesirable changes in health behaviours among young adults [20]. A prospective study of 291 young adults who were followed from their final year of high school until their second year of university (college) found that students gained, on average, 2.7 kg during this transitional period [20]. Lower sport participation rates, higher internet use, and lower studying rates were associated with greater weight gain [20]. Moreover, low levels of physical activity and poor dietary habits have been observed among university students in health-related disciplines where one may expect them to model healthful behavioural patterns [21]. Importantly, the adoption of healthful behaviours including regular physical activity and consumption of nutrient-rich foods in young adulthood has been associated with weight maintenance and a lower risk of developing chronic disease in later adult life [22]. Therefore, the establishment and maintenance of healthful behavioural patterns during this transitional life stage is critical to preventing weight gain and associated chronic disease risk.

Recent reviews have highlighted the limited evidence base for successful weight management (i.e., prevention of weight gain) interventions targeting young adults [23–31], with large heterogeneity in intervention design and outcomes observed [25, 27–29, 31]. Young adulthood is a developmentally unique life stage [6]. Consequently, interventions aimed at this demographic must address the factors known to contribute to weight gain during this transitional period [6] and work to remove the perceived and actual barriers to adopting more healthful behavioural patterns [32]. For young adults, barriers to weight management often exceed enablers [32], with healthful eating and physical activity not considered high priorities [33]. Young adults are typically less concerned with their future health and wellbeing, and as a result, engage in more risky health behaviours [34]. Perceived time constraints, busy schedules, work demands, lack of discipline, inadequate self-regulation skills, and a lack of environmental support for healthful eating and physical activity have been cited as barriers to weight management [32, 33, 35–37]. Similarly, enablers to weight management include knowledge and awareness, self-regulation skills, self-efficacy, and social and environmental support [32, 33, 36, 38]. Comprehensive empirical investigations of the relative importance of individual, social, and environmental barriers and enablers among young adults are lacking. Further research is needed to inform the systematic development of weight gain prevention interventions that can be implemented to effectively promote the adoption and maintenance of healthful behavioural patterns in young adults by removing identified barriers and promoting enablers.

Understanding health behaviours, and the settings in which they occur, is requisite for the design of effective and efficient behavioural interventions [39, 40]. Building an evidence base that can be reliably drawn upon to deliver beneficial change across different behaviours, populations, and contexts relies on the rigorous application and reporting of theory by researchers and practitioners in the design, implementation, and evaluation of interventions [41]. Findings from a recent systematic review [30] of reported theory use in weight management intervention targeting young adults revealed a majority of interventions do not rigorously apply theory. While many studies made mention of theory, few integrated the referenced theory (or theories) throughout all intervention stages. Of note, the lack of studies explicitly linking theoretical constructs to target behaviours in intervention design [42] limits understanding of which constructs elicit (or not) the desired change in young adult’s weight-related behaviours [43] and their associated outcomes. In addition, review findings revealed only a small number of theories had been applied in intervention design, namely *Social Cognitive Theory (SCT)* [44], *Self-Determination Theory (SDT)* [45], and the *Theory of Reasoned Action/Planned Behaviour (TRA/TPB)* [46, 47]. Although there is evidence to support the explanatory and/or predictive capacity of these predominantly individual-focused social psychological theories (e.g. TRA/TPB), albeit with varying degrees of success [48, 49], an over-reliance on the most popular theories without direct empirical tests or questioning of underlying assumptions limits progress in the field [50–52].

Many popular theories of behaviour focus on the intra-individual, and occasionally interpersonal factors, of behaviour while failing to account for the complex social and physical environments in which behaviour occurs [40]. Moreover, many popular theories of behaviour (e.g. TRA/TPB) focus on the rational drivers of individual behaviour and ignore the important roles of automaticity, impulsivity, habit, self-control, associative learning, and emotional processing [53]. Weight-related behaviours are determined by a complex interplay between individual, social, and environmental factors; therefore, theories selected to inform intervention design in this context must provide a systematic means of understanding behaviour and be capable of capturing the full range of potential levers of change [40].

Taken together, the small number of mainly individual-focused social psychological theories being applied in this context, coupled with a general lack of rigorous theory application, is likely to be limiting the effectiveness of weight gain prevention interventions targeting young adults. The purpose of this study is to systematically identify enablers and barriers for two weight-related behaviours (eating and physical activity) among young adults using the COM-B model [54].

### Theoretical framework

**Fig. 1:**
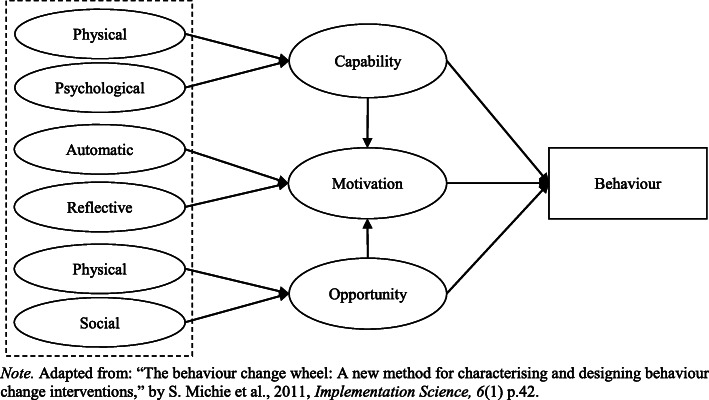
The COM-B model

The COM-B model [54] conceptualises behaviour as part of a system of interacting factors. According to the COM-B model, for a given behaviour to occur, at a given moment, one must have the capability and opportunity to engage in the behaviour, and the strength of motivation to engage in the behaviour must be greater than for any other competing behaviour [40]. As illustrated in Fig. 1, capability, opportunity, and motivation interact to generate behaviour that may, in turn, affect these factors.

Capability is defined as an individual’s psychological and physical capacity to engage in the behaviour concerned. Physical capability relates to whether an individual possesses the necessary knowledge and skills required to perform the target behaviour. Psychological capability refers to an individual’s capacity to engage in the necessary thought processes, comprehension, and reasoning to perform the target behaviour [54]. Research has shown capability to be associated with motivation and behaviour in a range of contexts [55–60]. In the COM-B model, capability is said to be associated with behaviour directly and indirectly via the mediating effect of motivation (see Fig. 1).

Opportunity is defined as all the external factors that lie outside an individual that make behaviour possible or prompt it, including physical and social factors [54]. Physical opportunity is afforded by the built environment and social opportunity is afforded by the cultural milieu that dictates the way that individuals think about things [54]. Past research suggests the role of opportunity in directly influencing behaviour is variable. Some studies have shown opportunity to be associated with behaviour [56, 58, 60]; whereas, other studies have reported no correlation between opportunity and behaviour [55, 57]. Of note, research has found that if both the social and physical environment are supportive of healthful eating and physical activity individuals will be more likely to engage in these behaviours [61]. Moreover, a prospective cohort study by Howlett et al. [59] found (social) opportunity to have an indirect association with physical activity via the mediating effect of motivation in an adult sample. An additional study by Howlett et al. [58] found opportunity to directly predict motivation and sitting behaviour. In the COM-B model, opportunity is said to be associated with behaviour directly and indirectly via the mediating effect of motivation (see Fig. 1).

Motivation is defined as all the brain processes that energise and direct behaviour, not just goals and conscious decision-making [54]. A distinction is made between reflective motivational processes (evaluations and plans) and automatic motivational processes (emotions and impulses) [54]. Past research has shown motivation to be associated with behaviour in a range of contexts [55–57, 59, 60, 62]. Of note, Moorman and Matulich [62] found motivation to be independently correlated with preventive health behaviours. Similarly, Howlett et al. [59] found motivation to be directly associated with physical activity behaviour in adults and partially mediate associations between capability, opportunity, and behaviour. In contrast, Howlett et al. [58] found no direct association between motivation and sitting behaviour. In the COM-B model, motivation is said to be directly associated with behaviour and is proposed to mediate the associations between capability, opportunity, and behaviour (see Fig. 1).

Behaviour is defined as any action a person takes in response to internal or external events [39]. Actions may be overt and directly measurable or covert and indirectly measurable [39]. In line with Australia’s Physical Activity & Sedentary Behaviour Guidelines for Adults (18–64 years), young adults should be active on most, preferably all, days of the week, accumulating 150 to 300 min (2.5 to 5 h) of moderate intensity activity or 75 to 150 min (1.25 to 2.5 h) of vigorous intensity activity, or an equivalent combination of both each week [63]. In addition, they should complete muscle strengthening activities on at least 2 days each week. In terms of sedentary behaviour, young adults should minimise the amount of time spent in prolonged sitting and break up long periods of sitting as often as possible. According to Australian Dietary guidelines, young adults should consume fruit and vegetables, legumes, wholegrains, reduced fat milk, yoghurt, and cheese varieties, lean meats and poultry, fish, eggs, nuts, and seeds for a nutrient-rich diet, and drink water instead of sugar sweetened beverages and alcoholic drinks [64].

Behavioural interventions targeting weight management in young adults are often complex encompassing multiple components (e.g., behavioural change techniques and technologies) and targeting multiple behaviours [29, 30] that interact together to effect weight status as measured by body mass index (BMI). Physical inactivity and unhealthy dietary habits (i.e., consumption of energy-dense, nutrient-poor foods) are two behaviours most associated with weight status [18, 22, 65] and are modifiable risk factors of chronic disease [66]. Weight management is defined as the prevention of weight gain through the maintenance of a healthy body weight or the reversal of small gains to maintain a healthy body weight [67]. Even small changes in physical activity and dietary patterns can contribute to weight management owing to desired shifts in energy balance (e.g., energy expenditure is equal, or greater than, energy intake). For example, research suggests that small changes in behaviour that amount to a decrease in calorie intake of only 100 cal per day can effectively prevent weight gain [68]. Randomised controlled trials of behavioural interventions have reported moderate success in changing weight-related behaviours in prevention trials [23, 25, 69] and clinically significant reductions in overweight and obesity [70, 71]. Identifying and understanding the active ingredients within behavioural interventions is key to achieving desired outcomes.

In the present study, we operationalise and test the COM-B model to examine the role of capability, opportunity, and motivation in young adult’s eating and physical activity behaviours. We hypothesise that capability, opportunity, and motivation will be associated with young adult’s physical activity and eating behaviours. In addition, we hypothesise that capability and opportunity will be indirectly associated with behaviour via the mediating effect of motivation. Lastly, we hypothesise that capability, opportunity, and motivation will explain young adult’s physical activity and eating behaviours.

## Methods

### Procedure

The research protocol for this study was approved by the Human Research Ethics Committee at the principal investigator’s institution before study commencement (ref no. 2017/308). A cross-sectional survey research design was employed to empirically test the COM-B model in the contexts of young adult’s eating and physical activity behaviours. Both surveys (physical activity and eating) were developed and administered online using LimeSurvey. The physical activity survey was disseminated in April 2018 and the eating survey was disseminated in October 2018. The landing page of both surveys included the purpose of the research, approximate duration of the survey, incentive for participation, notification of the voluntary nature of participation, and a link to the full information sheet. By clicking continue, participants consented to their participation. Respondents who completed the survey in its entirety were given the option to enter a random draw to win one of 10 $50 gift vouchers. The use of a prize draw as an incentive for participation was outlined in the approved research protocol. Participation in the study was entirely voluntary and participants could withdraw from the study at any point without repercussions. Research supports the use of incentives (e.g., random prize draw) [72, 73] and there is no conclusive evidence to suggest the use of incentives results in coercion [74]. To further optimise survey response rates and reduce respondent burden, a staggered delivery (see dissemination timeline above) was employed. Participants who completed the physical activity survey and agreed to be contacted for future research were sent invitations to participate in the eating survey. In accordance with best practice, reminder messages were sent to participants to complete the eating survey [75].

### Participants

The target population of interest for this study was Australian adults aged 18–35 years. A combination of convenience, purposive, and snowball sampling methods was used to recruit eligible participants. The use of multiple sampling techniques was expected to increase survey response rates [76]. In Australia, 88% of university students (including associate, undergraduate, and postgraduate degrees) fall within the 18–35-year age range [77]. Student and staff broadcast emails were used to recruit eligible participants from six large Australian universities. Snowball sampling was also initiated by encouraging respondents who completed the survey to share the survey link via their social networks. Participants were screened for eligibility (via nationality and date of birth) prior to inclusion.

### Measures

Pre-validated measures (indicators) appropriate for capturing the latency of COM constructs in the contexts of physical activity and eating were sourced from the literature to create two survey instruments. Measures were informed by the *Theoretical Domains Framework (TDF)* [78], with domains mapped onto COM constructs (see Additional File 1). This method has been applied in previous research [58, 59]. Where possible, the same measures were used in both surveys, with minor adaptions made to item wording to match behavioural context. Both survey instruments were developed for specific use in this study (see Additional File 2). For instances where measures could not be adapted (e.g., environmental context and resources), a further literature search was performed to source contextually appropriate measures. A total of 17 pre-validated measurement scales were obtained. The physical activity survey consisted of 137 items and the eating survey consisted of 120 items. Five- and seven-point scales (unipolar, bipolar, and semantic differential) were used.

In terms of outcome measures, physical activity was measured using the *International Physical Activity Questionnaire Short Form (IPAQ-SF)* scale [79]. The 9-item IPAQ-SF is a widely accepted measure of self-reported physical activity that has been shown to be reliable and valid [79, 80]. The IPAQ-SF records activity across four levels: 1) vigorous-intensity activity, 2) moderate-intensity activity, 3) walking, and 4) sitting. A metabolic equivalent of task (MET) score is calculated for each activity type by weighting its energy requirements: 3.3 METs for walking, 4 METs for moderate-intensity activity, and 8 METs for vigorous-intensity activity. A total moderate to vigorous physical activity (MVPA) MET score is calculated from the sum of MET-minutes/week score for each level. Participants may overestimate their self-reported physical activity when compared to objective measures [81]; a common limitation of subjective measures. A 7-day recall of activity levels was used in the current study to reduce respondent burden [79]. Diet was measured using 12 items derived from a previously validated measurement instrument that assesses food group consumption frequency in line with the Dietary Guidelines for Australian Adults [82–84]. Each item is scored from 0 to 10 to reflect compliance with dietary guidelines for that specific food group [64] and then summed to create a total score ranging from 0 to 120 (higher scores indicate better diet quality).

To capture sample characteristics, several socio-demographic questions were asked at the end of the survey. Socio-demographic variables included: age, sex, education, employment, income, relationship status, and ethnicity. Socio-demographic variables were used for descriptive purposes only (see Additional File 3).

### Analysis

Descriptive statistics were analysed using SPSS v25. Exploratory factor analyses were performed in SPSS to ascertain the underlying structure of the measures selected to capture the latent COM constructs. Confirmatory factor analyses were conducted in AMOS v25. Anderson and Gerbing’s [85] two-step approach to structural equation modelling was then followed. Both measurement and structural models were specified and tested in AMOS v25 using maximum likelihood estimation. A final structural model was established through a stepwise process of model trimming: (i) removing statistically non-significant (*p* > .05) indicators; and (ii) removing indicators with low squared multiple correlations (SMC < .3). Several goodness-of-fit indices were used to determine model acceptability (see Table 1).

**Table 1:** Goodness-of-fit indices and their associated cut-offs

Index	Acceptable fit	Good fit	Source
CMIN/DF	> 2.00 but ≤ 3.00	< 2.00	[86]
RMSEA	> .05 but ≤ .08	≤ .05	[87]
SRMR	> .05 but ≤ .10	≤ .05	[88]
CFI	> .95	> .97	[86]
TLI	> .95	> .97	[86]
GFI	> .90	> .95 but ≤ 1.00	[86]
AGFI	> .90	> .95 but ≤ 1.00	[86]

*Note.* acceptable fit indices used

Abbreviations: *CMIN/DF* normed chi-square, *RMSEA* root mean square error of approximation, *SRMR* standardised root mean square residual, *CFI* comparative fit index, *TLI* Tucker-Lewis index, *GFI* goodness-of-fit index, *AGFI* adjusted goodness-of-fit index

## Results

### Sample characteristics

#### Physical activity survey

The physical activity survey received a total of 1104 initial responses, 275 of which were deemed to be non-responses (< 1 measure completed). A further 247 responses were deleted owing to significant item non-response, leaving a total usable sample of 582 cases. Cases with a significant percentage (missing rate > 10%) of item nonresponse were removed as statistical analysis is likely to be biased when more than 10% of data are missing [89]. The mean age of participants in the sample was 22.8 years (SD = 4.87), with a large proportion being Caucasian (82.5%), female (80.3%), full-time university students (57.7%) earning $75,000 or less a year (77.9%). The mean BMI among participants was 20.30 (SD = 4.60). See Additional File 3 for sample characteristics.

#### Eating survey

The eating survey received a total of 805 initial responses, 234 of which were deemed to be non-responses (< 1 measure completed). A further 116 responses were deleted owing to significant item non-response [89], leaving a total usable sample of 455 cases. The mean age of participants in the sample was 24.9 years (SD = 5.15), with a large proportion being Caucasian (72.5%), female (80.8%), full-time university students (52.2%) earning $75,000 or less a year (59.3%). The mean BMI among participants was 24.21 (SD = 5.65). See Additional File 3 for sample characteristics.

#### Levels of physical activity and diet quality scores

Most participants (71.5%) reported completing some level (vigorous, moderate, or walking) of activity every day of the week. Average time (total in minutes) spent being physically active (vigorous, moderate, and walking) during a given week was 152.48 (SD = 91.91). Average time (total in minutes) spent engaging in MVPA was 90.79  (SD = 71.46). The mean diet quality score was 78.03 (SD = 15.13). Levels of physical activity and dietary quality scores reflect broader trends observed among young adults [9, 11, 16], and in particular, university students [8].

### Descriptive statistics

Following reliability analyses, composite measures were created as a sum of the total divided by the number of items in the measurement scale [90]. Table 2 presents the descriptive statistics of all composite measures created.

**Table 2:** Descriptive statistics of composite measures

	Measure	Physical activity					Eating				
		*N* of items	*N*	Min.	Max.	M (SD)	*N*	*N* of items	Min.	Max.	M (SD)
*Capability*	Knowledge (score)	6	582	0	6	4.90 (1.06)	455	8	0	8	6.75 (1.44)
	Perceived competence	5	582	1	7	3.96 (1.39)	455	7	1	7	4.99 (1.34)
	Decision making (pros)	9	582	1	7	6.06 (.86)	455	4	1	7	6.31 (.80)
	Decision making (cons)	3	582	1	7	3.42 (1.43)	455	5	1	7	3.45 (1.17)
	Action control	5	582	1	7	4.28 (1.70)	455	6	1	7	4.70 (1.30)
	Action planning	4	582	1	7	4.27 (1.94)	455	4	1	7	3.65 (1.80)
	Habits	12	582	1	7	3.81 (1.56)	455	11	1	7	4.37 (1.45)
*Opportunity*	Subjective norms	5	582	1	7	3.95 (1.24)	455	–	–	–	–
	Subjective norms (injunctive)	–	–	–	–	–	455	3	1	7	4.60 (1.41)
	Subjective norms (descriptive)	–	–	–	–	–	455	3	1	7	4.42 (1.08)
	Social support	10	582	1	5	2.49 (.40)	–	–	–	–	–
	Social support (positive)	–	–	–	–	–	455	3	1	5	2.82 (1.15)
	Social support (negative)	–	–	–	–	–	455	4	1	5	2.24 (1.01)
	Perceived environment (PA)	5	582	1	5	3.75 (.85)	–	–	–	–	–
	Perceived environment (home)	–	–	–	–	–	455	4	1	7	5.50 (1.13)
	Perceived environment (shop)	–	–	–	–	–	455	3	1	7	5.57 (1.04)
	Perceived environment (work)	–	–	–	–	–	455	3	1	7	4.28 (1.46)
	Resources (score)	8	582	0	8	5.05 (2.04)	–	–	–	–	–
*Motivation*	Identity	9	582	1	7	4.15 (1.68)	455	3	1	7	5.47 (1.12)
	Self-efficacy	3	582	1	7	5.06 (1.57)	455	3	1	7	5.29 (1.26)
	PBC	3	582	1	7	5.78 (1.33)	455	3	1	7	5.99 (1.02)
	Attitudes (instrumental)	3	582	1	7	6.33 (1.22)	455	3	1	7	6.62 (.81)
	Attitudes (affective)	3	582	1	7	5.34 (1.57)	455	3	1	7	5.43 (1.33)
	Intentions	3	582	1	7	4.84 (1.76)	455	5	1	7	5.90 (.92)
	Goals (social)	4	582	1	7	3.58 (1.73)	–	–	–	–	–
	Goals (appearance)	3	582	1	7	5.51 (1.49)	–	–	–	–	–
	Goals (personal)	4	582	1	7	5.09 (1.49)	–	–	–	–	–
	Goals (eating)	–	–	–	–	–	455	6	1	5	3.37 (.95)
	Goals (drinking	–	–	–	–	–	455	4	1	5	2.84 (1.09)
	Reinforcement	2	582	1	5	2.71 (1.02)	455	–	–	–	–
	Reinforcement (control)	–	–	–	–	–	455	4	1	7	4.16 (1.49)
	Reinforcement (mind)	–	–	–	–	–	455	3	1	7	3.25 (1.75)
	Affect (positive)	5	582	1	5	3.53 (.88)	455	2	1	5	3.18 (1.05)
	Affect (negative)	5	582	1	5	2.07 (.88)	455	4	1	5	2.16 (.99)

*Note.* Composite measures were created as a sum of the total divided by the number of items in the measurement scale. Where required, items with low item-total correlations were removed from scales to improve reliability

Abbreviations: *PBC* perceived behavioural control

### Scale reliability and validity

All measurement scales had acceptable item-total correlations (> .30). All measurement scales had Cronbach’s Alpha (*α*) values > .70 [90], with the exception of descriptive norms (see Table 3). Convergent validity was evaluated using standardised regression weights: loadings that were significant and greater than 0.5 (ideally > .70) were considered valid. These findings were confirmed by examining the average variances extracted (> .50) [91]. Discriminant validity was assumed if average variance extracted estimates were greater than the squared correlation estimates [91], see Table 4. Composite reliability was calculated as an additional measure of the shared variance among the observed variables (i.e., indicators of latent COM constructs) with a threshold of > .70 used [91], refer to Table 4.

**Table 3:** Internal consistency of measurement scales

Measure	Physical activity		Eating	
	No. of items	*α*	No. of items	*α*
Perceived competence	5	.87	7	.87
Decision making (pros)	9	.91	4	.82
Decision making (cons)	3	.80	5	.77
Action control	5	.91	6	.88
Action planning	4	.95	4	.89
Habits	12	.97	11	.96
Subjective norms	5	.73	–	–
Injunctive norms	–	–	3	.85
Descriptive norms	–	–	3	.65
Social support	10	.92	–	–
Social support (negative)	–	–	4	.84
Social support (positive)	–	–	3	.87
Perceived environment (PA)	5	.85	–	–
Perceived environment (home)	–	–	4	.78
Perceived environment (shop)	–	–	3	.76
Perceived environment (work)	–	–	3	.82
Identity	9	.95	3	.77
Self-efficacy	3	.90	3	.87
PBC	3	.87	3	.85
Attitudes (instrumental)	3	.89	3	.80
Attitudes (affective)	3	.89	3	.89
Intentions	3	.96	5	.91
Goals (social)	4	.93	–	–
Goals (appearance)	3	.95	–	–
Goals (personal)	4	.89	–	–
Goals (eating)	–	–	6	.84
Goals (drinking)	–	–	4	.71
Reinforcement	2	.80	–	–
Reinforcement (control)	–	–	–	.82
Reinforcement (mind)	–	–	–	.91
Affect (positive)	5	.91	2	.76
Affect (negative)	5	.88	4	.83

*Note.* Abbreviations: *PBC* perceived behavioural control

**Table 4:** Discriminant validity

	Physical activity							Eating				
	*N*	AVE	CR	1.	2.	3.	*N*	AVE	CR	1.	2.	3.
1. Capability	3	.80	.92		C: .55 C^2^: .30	C: .97 C^2^: .94	3	.67	.86		C: .82 C^2^: .67	C: .94 C^2^: .90
2. Opportunity	2	.78	.88			C: .61 C^2^: .37	3	.54	.78			C: .77 C^2^: .60
3. Motivation	4	.69	.90				6	.59	.90			

*Note.* Values calculated from trimmed measurement model

Abbreviations: *AVE* average variance extracted, *C* correlation, *C*^2^ correlation squared score, *CR* composite reliability, *N* number of indicator variables

### Measurement and structural model validation

Several statistically unreliable indicators (SMC < .30; *p* > .05) were removed from the physical activity model one at a time, leaving a fully trimmed model with only statistically significant (*p* < .05) indicators. The final physical activity measurement model (see Additional File 4) comprised three latent constructs and nine composite measures (indicators). Fit indices indicated structural validity: χ^2^ (28.584)/df (21) = 1.361; GFI = .989, AGFI = .977; CFI = .998; TLI = .996; RMSEA = .025; SRMR = .014.

Specification of the eating measurement model followed the same process with several statistically unreliable indicators (SMC < .30; *p* > .05) removed from the model one at a time, leaving a fully trimmed model with only statistically significant (*p* < .05) indicators. The final eating measurement model (see Additional File 5) comprised three latent constructs and 12 composite measures (indicators). Fit indices indicated structural validity: χ^2^ (72.402)/df (37) = 1.957; GFI = .974, AGFI = .945; CFI = .987; TLI = .976; RMSEA = .046; SRMR = .026.

**Fig. 2:**
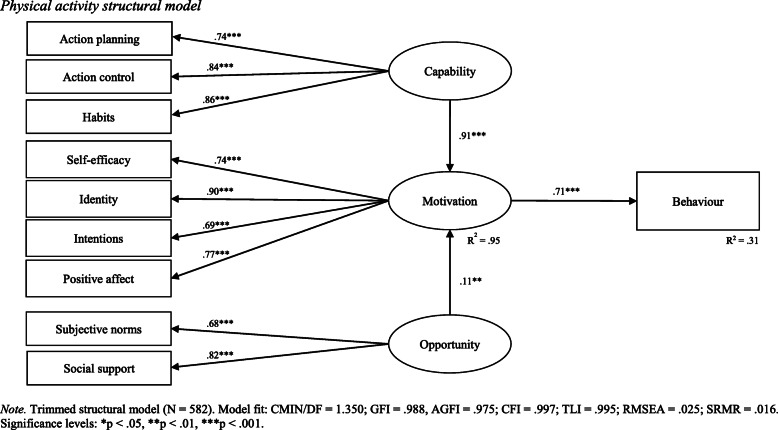
Physical activity structural model

Following measurement model validation, a physical activity structural path model was specified and tested. Stepwise theoretically supported adjustments were made to the structural model according to modification indices. Of note, the direct paths between capability and behaviour, and opportunity and behaviour, were removed. Only indirect associations via motivation remained in the final structural model. The final physical activity structural model demonstrated acceptable fit with sample data (CMIN/DF = 1.350; GFI = .988; AGFI = .975; CFI = .997; TLI = .995; RMSEA = .025; SRMR = .016). The results of the final physical activity structural model are reported in Table 5 and visually depicted in Fig. 2.

**Table 5:** Physical activity structural model results

		*β*	S.E.	C.R (t)	*p*
Behaviour	Motivation	.71	.03	15.52	.001
Motivation	Capability	.91	.05	20.84	.001
Motivation	Opportunity	.11	.07	3.09	.002
Habits	Capability	.86	.05	19.60	.001
Action control	Capability	.84	.04	25.36	.001
Action planning	Capability	.74	.05	19.60	.001
Social support	Opportunity	.82	.08	11.11	.001
Subjective norms	Opportunity	.68	.10	11.11	.001
Identity	Motivation	.90	.07	19.60	.001
Intentions	Motivation	.69	.04	19.54	.001
Positive affect	Motivation	.77	.02	23.70	.001
Self-efficacy	Motivation	.74	.04	19.62	.001

*Note.* Regression weights are standardised. Total variance explained by the indicators of motivation was 95%. Motivation explained 31% of the variance in level of physical activity

As shown in Table 5 (and Fig. 2), variance explained in physical activity behaviour (as measured via IPAQ-SF scores) was 31%. Motivation was positively associated with physical activity behaviour (*β* = .71, *p* < .001). This means the higher young adult’s motivation is to be physically active, the higher their level of physical activity. Equally, the lower young adult’s motivation is to be physically active, the lower their level of physical activity. The four statistically significant indicators of motivation were identity (*β* = .90, *p* < .001), positive affect (*β* = .77, *p* < .001), self-efficacy (*β* = .74, *p* < .001), and intentions (*β* = .69, *p* < .001). All four indicators had a positive association with motivation to be physically active.

Capability was positively associated with physical activity behaviour via the mediating effect of motivation (*β* = .91, *p* < .001). Consequently, the more capable young adults are (or feel they are) in being physically active, the more motivated they will be to engage in physical activity, thereby resulting in higher levels of physical activity. Similarly, the less capable young adults are (or feel they are) in being physically active, the less motivated they will be to engage in physical activity, thereby resulting in lower levels of physical activity. The three statistically significant indictors of capability were habits (*β* = .86, *p* < .001), action control (*β* = .84, *p* < .001), and action planning (*β* = .74, *p* < .001). All three indicators had a positive association with capability to be physically active.

Opportunity was positively associated with physical activity behaviour via the mediating effect of motivation (*β* = .11, *p* < .001). Thus, an environment conducive to physical activity increases motivation to be physically active, and in turn, results in higher levels of physical activity. In contrast, an environment that does not support physical activity decreases motivation to engage in physical activity, and in turn, results in lower levels of physical activity. The two statistically significant indicators of opportunity were social support (*β* = .82, *p* < .001) and subjective norms (*β* = .68, *p* < .001). Both indicators had a positive association with opportunity to be physically active.

**Fig. 3:**
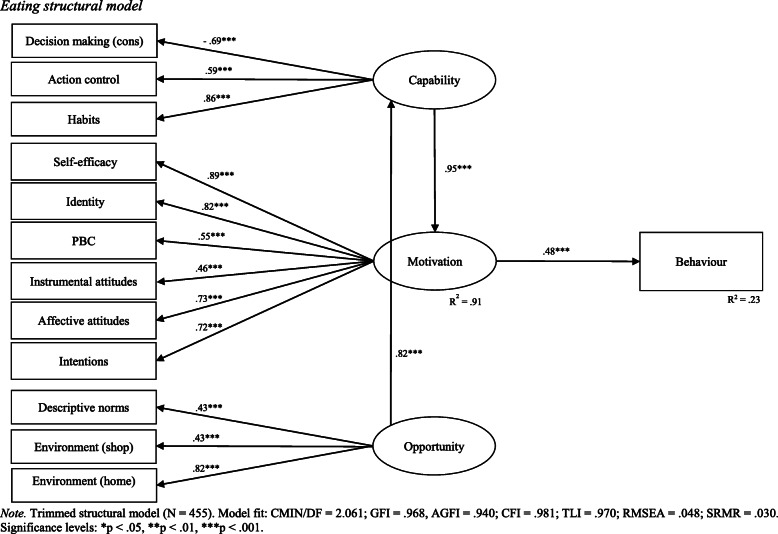
Eating structural model

Specification of the eating structural path model followed the same process as the physical activity model. Following measurement model validation, a structural path model was specified and tested. Stepwise theoretically supported adjustments were made to the structural model according to modification indices. Of note, the direct paths between capability and behaviour, and opportunity and behaviour, were removed. Only indirect associations via motivation remained in the final structural model. In addition, opportunity was found to be associated with motivation via the mediating effect of capability. The direct path between opportunity and motivation was subsequently removed. The final eating structural model demonstrated acceptable fit with sample data (CMIN/DF = 2.061; GFI = .968, AGFI = .940; CFI = .981; TLI = 0.970; RMSEA = 0.048; SRMR = .030). The results of the final eating structural model are reported in Table 6 and visually depicted in Fig. 3.

**Table 6:** Eating structural model results

		*β*	S.E.	C.R (t)	*p*
Behaviour	Motivation	.48	.77	10.42	.001
Motivation	Capability	.95	.04	19.01	.001
Capability	Opportunity	.82	.10	10.74	.001
Habits	Capability	.86	.12	13.41	.001
Decision making (cons)	Capability	−.69	.04	−16.66	.001
Action control	Capability	.59	.05	13.41	.001
Environment (home)	Opportunity	.82	.26	7.94	.001
Subjective norms (descriptive)	Opportunity	.43	.06	7.97	.001
Environment (shop)	Opportunity	.43	.06	7.94	.001
Identity	Motivation	.82	.07	19.78	.001
Intention	Motivation	.72	.04	19.78	.001
Attitudes (affective)	Motivation	.73	.06	16.43	.001
Attitudes (instrumental)	Motivation	.46	.04	9.20	.001
Perceived behavioural control	Motivation	.55	.06	11.02	.001
Self-efficacy	Motivation	.89	.06	19.73	.001

*Note.* Regression weights are standardised. Total variance explained for motivation was 91%. Motivation explained 23% of the variance in eating behaviour

As shown in Table 6 (and Fig. 3), variance explained in eating behaviour (as measured via diet quality score) was 23%. Motivation was positively associated with eating behaviour (*β* = .48, *p* < .001). Thus, the more motivated young adults are to eat healthily, the higher their diet quality will be. Conversely, the less motivated young adults are to eat healthily, the lower their diet quality will be. The six statistically significant indicators of motivation were self-efficacy (*β* = .89, *p* < .001), identity (*β* = .82, *p* < .001), affective attitudes (*β* = .73, *p* < .001), intention (*β* = .72, *p* < .001), perceived behavioural control (*β* = .55, *p* < .001), and instrumental attitudes (*β* = .46, *p* < .001). All six indicators were positively associated with motivation to eat healthily.

Capability was positively associated with eating behaviour via the mediating effect of motivation (*β* = .95, *p* < .001). Thus, the more capable young adults are (or believe they are) in eating healthily, the more motivated they will be to consume nutrient-rich foods, and in turn, the higher their diet quality will be. Conversely, the less capable young adults are (or believe they are) in eating healthily, the less motivated they will be to consume nutrient-rich foods, and in turn, the lower their diet quality will be. The three statistically significant indictors of capability were habits (*β* = .86, *p* < .001), decision making cons (*β* = −.69, *p* < .001), and action control (*β* = .59, *p* < .001). Habits and action control were positively associated with capability to eat healthily, whereas decision making (cons) was negatively associated with capability to eat healthily.

Opportunity was positively associated with motivation via the mediating effect of capability (*β* = .82, *p* < .001). This means the more conducive an environment is to healthy eating, the more capable young adults are (or feel they are), and in turn, the more motivated they are to eat healthily. The three statistically significant indictors of opportunity were home environment (*β* = .82, *p* < .001), descriptive norms (*β* = .43, *p* < .001), and shop environment (*β* = .43, *p* < .001). All three indicators had a positive association with opportunity to eat healthily.

## Discussion

### Main findings

The purpose of this study was to systematically identify enablers and barriers for eating and physical activity behaviours among young adults using the COM-B model [57]. Findings revealed the COM-B model’s explanatory potential to vary across behavioural contexts (R^2^ = 0.31 for physical activity and R^2^ = 0.23 for eating). The variance explained by both models compares favourably to that of other social psychological theories [49, 92–94]. Variance explained has been reported to be between 14 and 24% for the TPB [49]. Conversely, a review summarising multiple social psychological theories (TPB, SCT, SDT, HPM, and TTM) reported explained variance in adolescent’s physical activity behaviours to range from 24 to 35% [94], indicating that the COM-B model, as measured and tested in the present study, can offer an alternate theoretical perspective (socio-ecological) explaining between 23 and 31% of young adult’s eating and physical activity behaviours. In practical terms, the COM-B model can identify individual, social, and environmental factors enabling and/or preventing healthy eating and physical activity in young adults.

In the physical activity model, capability and opportunity were found to be positively associated with physical activity behaviour through the mediating effect of motivation. In the eating model, capability was found to be associated with behaviour through the mediating effect of motivation, with capability mediating the association between opportunity and motivation. Previous research has reported similar interactions with motivation mediating the effect of opportunity and ability (capability) on energy saving behaviours [60]. The strongest indicators of COM constructs in the physical activity model were *habits* (capability), *social support* (opportunity), and *identity* (motivation). In the eating model, *habits* (capability), *home environment* (opportunity), and *self-efficacy* (motivation) were the strongest indicators of COM constructs. Taken together, findings confirm that weight-related behaviours such as eating and physical activity are associated with a complex combination of an individual’s capabilities, opportunities, and motivations. Interventions must, therefore, be designed to encompass the full range of enablers and barriers  (i.e., levers of change) identified.

### Implications for research

Interventions within the context of weight management often target multiple behaviours. For example, a recent review [29] found the reported behavioural focus of weight management interventions targeting young adults varied considerably with 29% of interventions reportedly targeting multiple behaviours (e.g. eating, physical activity, stress, and sleep). A further, 42% of interventions reportedly targeted both eating and physical activity. Unpacking such complex interventions is critical in understanding how they are (or are not) achieving desired outcomes. Theory can be applied to deconstruct interventions and identify underlying mechanisms of action [39, 95]; however, research has revealed a general lack of rigorous theory application in the context of weight management in young adults, with only a small number of mainly individual-focused social psychological theories dominating intervention design [30]. Developing effective interventions for the prevention of weight gain during young adulthood necessitates a theoretical model that can capture the complexity of weight-related behaviours and permits the systematic identification of underlying mechanisms of action (i.e., enablers and barriers). Overall, findings support the COM-B model’s capacity to provide a socio-ecological perspective of young adult’s eating and physical activity behaviours.

Furthermore, findings support the COM-B model’s potential to address inherent limitations of dominant social psychological theories by incorporating both rational and non-rational intra-individual drivers of behaviour, interpersonal factors, and environmental factors. In so doing, the COM-B model demonstrates its capacity to capture the complexity of weight-related behaviours such as eating and physical activity. In addition, the method of measurement and analysis used in this study permitted mechanisms of action (i.e., enablers and barriers) underlying young adult’s eating and physical activity behaviours to be systematically identified within the COM-B model. These mechanisms of action (i.e., enablers and barriers) form potential targets for future intervention design.

Several consistencies and variations were observed across the eating and physical activity models in terms of the statistically significant indicators capturing latent COM constructs (see Table 7).

**Table 7:** Summary of consistencies and variations in COM indicators

COM-B Model		TDF domain	Measures	PA	E
Capability	Psychological	Behavioural regulation	Action control	✓	✓
		Behavioural regulation	Action planning	✓	✘
		Behavioural regulation	Habits	✓	✓
		Memory, attention, and decision processes	Decision making (cons)	✘	✓
Opportunity	Social	Social influences	Subjective norms (descriptive)	✓	✓
		Social influences	Social support	✓	✘
	Physical	Environmental context and resources	Environment (home/shop)	✘	✓
Motivation	Reflective	Social/professional role and identity’	Identity	✓	✓
		Beliefs about capabilities	Self-efficacy	✓	✓
		Beliefs about capabilities	PBC	✘	✓
		Intention	Intentions	✓	✓
		Beliefs about consequences	Attitudes (instrumental/affective)	✘	✓
	Automatic	Emotion	Positive affect	✓	✘

*Note.* consistencies and variations are based on final measurement model results

Abbreviations: *E* eating, *PA* physical activity, *PBC* perceived behavioural control

As shown in Table 7, action control and habits were the only consistent indicators of capability across models. In terms of variations, action planning was only statistically significant in the physical activity model, whereas decision making (cons) was only statistically significant in the eating model. This finding is a likely reflection of the habitual and often unplanned nature of eating behaviours [96] suggesting that for this specific sample perceived barriers to behavioural performance were more salient in the context of eating than in the context of physical activity. Action control, action planning, and habits are self-regulation processes that can facilitate behavioural enactment [97, 98]. Research indicates that habit formation is an effective behavioural regulation strategy that individuals can adopt to obtain goals and lessen cues from competing behaviours or temptations that can disrupt plans to engage in healthful behaviours such as physical activity and healthy eating [99, 100]. The negative association between decision making (cons) and capability is supported by evidence which indicates that if an individual perceives strong barriers to behavioural change, they are unlikely to act [48].

For opportunity, subjective norms were the only consistent indicator across models (see Table 7). In terms of variations, social support was only statistically significant in the physical activity model, whereas environment (home and shop) was only statistically significant in the eating model. Some studies have found subjective norms and health-related behaviours to be associated [101]; however, the evidence is mixed [49, 93]. Equally, associations between social support and physical activity behaviour have been observed [102–104]. Taken together, findings suggest that social support may be more salient in the context of young adult’s physical activity than in eating. In the eating model, home and shop environments were associated with opportunity. Thus, the more conducive our environment is to eating healthily, the more capable we are (or feel we are), and in turn, the more motivated we are to eat healthily. While accessibility of physical activity facilities can affect level of physical activity [105], descriptive statistics from the present study suggest environmental context and available resources were conducive to young adult’s physical activity. Thus, a lack of physical opportunity may not have been salient among this sample of young adults.

For motivation, identity, self-efficacy, and intentions were the only consistent indicators across models. In terms of variations, positive affect was only statistically significant in the physical activity model, whereas perceived behavioural control and attitudes (instrumental and affective) were only statistically significant in the eating model. Research suggests that self-identity plays a key role in motivational processes, intention formation, and behavioural enactment [106–110] in the context of physical activity [111, 112] and eating [113]. As such, young adults who assign themselves a strong identity for a given behaviour (e.g., exerciser or healthy eater) are likely to have stronger motivations to perform that behaviour and are subsequently more likely to enact the behaviour. Similarly, self-efficacy, perceived behavioural control, attitudes and intentions have all been associated with eating and physical activity behaviours [49, 92, 93, 114–117] and this link is partially supported by present study findings where young adults who believed they were capable, had control, held favourable attitudes, and/or intentions of being physically active or eating healthfully were more motivated to engage in the behaviour. Lastly, the presence of positive affect in the physical activity model suggests that young adults assign positive emotions (feelings) to physical activity. Thus, positive emotions associated with physical activity can elicit automatic motivational processes to engage young adults in the behaviour.

### Implications for practice

Study findings reveal several potential enablers and barriers to young adult’s eating and physical activity behaviours. The mapping of theoretical domains to the latent COM constructs in the current study permitted the operationalisation and testing of the COM-B model in the contexts of young adult’s eating and physical activity behaviours. Subsequently, key mechanisms of action (i.e., enablers and barriers) underlying young adult’s eating and physical activity behaviours were able to be systematically identified. These mechanisms of action represent potential targets for future interventions aiming to promote healthy eating and physical activity in young adults to support weight management. In the physical activity model, the strongest indicators of COM constructs were found to be habits (capability), social support (opportunity), and identity (motivation). Similarly, in the eating model, the main indicators of COM constructs were found to be habits (capability), home environment (opportunity), and self-efficacy (motivation). Future interventions seeking to positively change young adult’s healthy eating and physical activity behaviours could be designed and evaluated based on these identified mechanisms of action. For example, interventions could be built to deliver social support to increase motivation to engage in physical activity and/or design supportive food environments to encourage healthy eating. To increase both healthy eating and physical activity, interventions could target young adult’s peers, friends, and parents delivering positive changes to subjective norms. Habits were strong indicators of capability in both models; therefore, tactics such as exercise routines, magnets on fridges, smaller food serving ware, alerts, and other reminders could be embedded within intervention designs to prompt healthful behavioural changes.

Furthermore, intervention designers may test the efficiency of universal (e.g., targeting consistent indicators across contexts) versus tailored behavioural change strategies (e.g., targeting context-specific indicators) in terms of return on investment. For example, self-regulation processes (e.g., action control, action planning, and habit) were consistent indicators across models; therefore, interventionists could integrate strategies designed to support the planning, initiation, and maintenance of behaviour across both contexts. Conversely, physical environment was only statistically significant in the eating model, whereas social opportunity was consistent across models. Targeting both the social and physical environment may not be feasible. Government, infrastructure and urban design, or retailer actions needed to deliver environmental support and increase individual motivation to be physically active (e.g., provision of cycle and walking paths that are well lit) or eat healthfully (e.g., supermarket layout and food labelling) take substantial resource investment and time from initiation to implementation. Thus, selecting the most consistent factor (e.g., subjective norms) may be more economical.

Furthermore, understanding how capability and opportunity affect motivation, and in turn, behaviour is critical to optimising future intervention design. Findings support the notion that the greater capability and opportunity are, the more likely a behaviour is to occur because of the presence of motivation. In the absence of opportunity and capability, behaviour is unlikely to occur as motivation is likely to be lacking. Multicomponent interventions encompassing a range of behavioural strategies mapped to relevant COM constructs are needed; however, in the absence of replication studies to validate present study findings in different samples and settings, the effectiveness of an intervention designed based on identified mechanisms of action (i.e., enablers and barriers) cannot be guaranteed. This study represents an initial step toward the design of a weight management intervention that can effectively alter capabilities and opportunities to support desired behavioural patterns among young adults and ensure they are able to act on their motivations to be physically active and eat healthily.

### Limitations and future research directions

Several limitations, many of which offer directions for future research, are acknowledged. First, findings are drawn from two convenience samples of young adults limiting generalisability [23]. Of note, the sample was predominantly Caucasian, female, and tertiary educated. Consequently, findings are unlikely to be representative of the whole young adult population. Given research has shown obesity tends to be higher among women, minority racial/ethnic groups, and at lower socioeconomic status [118, 119], future research should aim to recruit a broader and more diverse sample of young adults.

Second, the self-selective nature of the study likely impacted the diversity of participants recruited [120] such that participants who were interested in physical activity and eating behaviours were more likely to be motivated to complete the survey resulting in self-selection bias. Research suggests women are generally more interested in health topics and exhibit more active information-seeking behaviour [121] which may explain why a large proportion of both samples were female. Males comprise only 20% of health behaviour research samples [122], contributing to a lack of evidence on how to increase their uptake of health promoting behaviours [123]. To improve generalisability, future research should employ tailored strategies to ensure a broader spectrum of gender identities are represented.

Third, the subjective nature of self-report measures may have biased findings. Self-report measures are associated with response bias, inattention, and socially desirable responding [124]. While measures were taken to minimise potential biases (e.g., use of pre-validated measurement scales, varied response formats, pilot testing, and staggered dissemination), objective measures can offer a more reliable and valid method of measurement [125]. Where possible, future research should incorporate objective measures.

Finally, the three explanatory constructs (capability, opportunity, and motivation in the COM-B model are not able to be directly observed and are therefore inferred from indicator variables. This study used a new method of measuring the three explanatory constructs in the COM-B model (mapping latent COM constructs to relevant TDF domains and sourcing indicators of each domain). While this method has been applied twice previously [58, 59], potential alternatives could be explored. Future research could explore the potential for an integrated COM-B model to establish whether this approach alleviates some of the challenges faced in operationalising and testing the COM-B model and/or whether this approach enhances the explanatory power of the model. Li et al. [60] offer an example using an integrated MOA framework. Establishing clear definitions, along with reliable and valid measures of the latent COM constructs, warrants further attention.

## Conclusions

The prevention of weight gain during young adulthood requires the establishment and maintenance of healthful behavioural patterns including healthy eating and physical activity. Identifying and understanding the full range of potential levers of change (i.e., enablers and barriers) are essential steps in the development of effective weight management interventions targeting young adults. Findings from the present study support the COM-B model’s explanatory potential in the contexts of young adult’s physical activity and eating behaviours. Barriers and enablers underlying young adult’s physical activity and eating behaviours were identified and represent potential targets for future intervention design. Further research is needed to validate present study findings across different populations and settings.

## Supplementary Information

**Additional file 1.** Mapping of COM constructs to TDF domains and associated measures. Supplementary table summarising the method of measuring latent COM constructs.

**Additional file 2.** Survey instruments. Supplementary tables comprising English-language copies of the survey instruments (questionnaires) used in this study.

**Additional file 3.** Sample characteristics for both surveys (eating and physical activity). Supplementary table  summarising key sample characteristics across datasets (eating and physical activity).

**Additional file 4.** Physical activity measurement model. Diagrammatic summary of physical activity measurement model.

**Additional file 5.** Eating measurement model. Diagrammatic summary of eating measurement model.

## Data Availability

Data available on request from the authors. Data that supports the findings of this study are available from the corresponding author (TJW) upon reasonable request. In accordance with the ethics approval granted for this study (ref no. 2017/308), data are not publicly available due to their containing information that could compromise the privacy of participants.

## Abbreviations

AGFI: Adjusted goodness-of-fit index
BMI: Body mass index
COM-B: Capability, Opportunity, Motivation, and Behaviour
CMIN/DF: Normed chi-square
CFI: Comparative fit index
E/HE: Eating / healthy eating
IPAQ-SF: International Physical Activity Questionnaire Short Form
GFI: Goodness-of-fit index
MET: Metabolic equivalent of task
MOA: Motivation, Opportunities, and Abilities
MOAB: Motivation, Opportunities, Abilities, and Behaviour
MVPA: Moderate to vigorous physical activity
NOA: Needs, Opportunities, Abilities
PA: Physical activity
PBC: Perceived behavioural control
RMSEA: Root mean square error of approximation
SCT: Social Cognitive Theory
SDT: Self-Determination Theory
SRMR: Standardised root mean square residual
TDF: Theoretical Domains Framework
TPB: Theory of Planned Behaviour
TRA: Theory of Reasoned Action
TLI: Tucker-Lewis index
