# Supplementary material for: Capability, opportunity, and motivation: an across contexts empirical examination of the COM-B model
Source: BMC Public Health. 2021 May 29;21:1014. doi: 10.1186/s12889-021-11019-w (PMC8164288; doi:10.1186/s12889-021-11019-w)
Supplement: Supplementary file 1 — Additional file 1. Mapping of COM constructs to TDF domains and associated measures. Supplementary table summarising the method of measuring latent COM constructs. [file 12889_2021_11019_MOESM1_ESM.pdf]

Additional File 1

Mapping of COM constructs to TDF domains and associated measures

			Physical activity		Eating	
	COM	TDF domain	Measure(s)	Source(s)	Measure(s)	Source(s)
Capability	Psychological	Knowledge	Physical activity knowledge	(1)	Nutrition knowledge	(2)
		Skills	Perceived competence	(3)	Perceived competence	(4)
		Memory, attention and decision processes	Decision making	(5)	Decisional balance	(6)
		Behavioural regulation	Action control<br>Action planning<br>Habits	(7)<br>(8)<br>(9)	Action control<br>Action planning<br>Habits	(7)<br>(8)<br>(9)
	Physical	Skills		-	-	-
Opportunity	Social	Social influences	Subjective norms<br>Social support	(10)<br>(11)	Subjective norms<br>Social support	(10, 12)<br>(6, 11)
	Physical	Environmental context and resources	Perceived environment	(13)	Perceived environment	(6, 14)
Motivation	Reflective	Social/professional role & identity	Identity	(15)	Identity	(16)
		Beliefs about capabilities	Self-efficacy<br>PBC	(10)	Self-efficacy<br>PBC	(10, 12)
		Optimism	-	-	-	-
		Beliefs about consequences	Attitudes (instrumental and affective)	(10)	Attitudes (instrumental and affective)	(10, 12)
		Intentions	Intentions	(10)	Intentions	(10, 12)
		Goals	Physical activity goals	(17)	Dietary goals	(18)
	Automatic	Social/professional role & identity	-	-	-	-
		Optimism	-	-	-	-
		Reinforcement	Reward-based exercise	(19)	Reward-based eating	(20)
		Emotion	Affect (positive and negative)	(21)	Emotional state	(22)

Table footnotes. Objective measurement of physical skills (PA and HE) was not possible; perceived competence was measured as a proxy. A valid measure of optimism (PA and HE) was not sourced. Abbreviations: HE = healthy eating; PA = physical activity; PBC = perceived behavioural control; TDF = theoretical domains framework.

Sources:

1. Rundle-Thiele, S., Kubacki, K., & Gruneklee, N. (2016). Perceived benefits and barriers of physical activity: A social marketing formative study. *Health Marketing Quarterly, 33*(2), 181-194. https://doi.org/10.1080/07359683.2016.1166872
2. Dickson-Spillmann, M., Siegrist, M., & Keller, C. (2011). Development and validation of a short, consumer-oriented nutrition knowledge questionnaire. *Appetite, 56*(3), 617-620. https://doi.org/10.1016/j.appet.2011.01.034
3. Fox, K. R., & Corbin, C. B. (1989). The physical activity self-perception profile: Development and preliminary validation. *Journal of Sport & Exercise Psychology, 11*(4), 408-430.
4. van der Horst, K., Brunner, T. A., & Siegrist, M. (2011). Ready-meal consumption: associations with weight status and cooking skills. *Public Health Nutrition, 14*(2), 239-245. https://doi.org/10.1017/S1368980010002624
5. Marcus, B. H., Rakowski, W., & Rossi, J. S. (1992). Assessing motivational readiness and decision making for exercise. *Health Psychology, 11(*4), 257-261. https://doi.org/10.1037/0278-6133.11.4.257
6. Norman, G. J., Carlson, J. A., Sallis, J. F., Wagner, N., Calfas, K. J., & Patrick, K. (2010). Reliability and validity of brief psychosocial measures related to dietary behaviors. *International Journal of Behavioral Nutrition and Physical Activity, 7*(1), 56-56. https://doi.org/10.1186/1479-5868-7-56
7. Luszczynska, A., & Schwarzer, R. (2003). Planning and self-efficacy in the adoption and maintenance of breast self-examination: A longitudinal study on self-regulatory cognitions. *Psychology & Health, 18*(1), 93-108. https://doi.org/10.1080/0887044021000019358
8. Sniehotta, F. F., Scholz, U., & Schwarzer, R. (2005). Bridging the intention-behaviour gap: Planning, self-efficacy, and action control in the adoption and maintenance of physical exercise. *Psychology & Health, 20*(2), 143-160. https://doi.org/10.1080/08870440512331317670
9. Verplanken, B., & Orbell, S. (2003). Reflections on past behavior: A self-report index of habit strength. *Journal of Applied Social Psychology, 33*(6), 1313-1330. https://doi.org/10.1111/j.1559-1816.2003.tb01951.x
10. Rhodes, R. E., & Courneya, K. S. (2003). Investigating multiple components of attitude, subjective norm, and perceived control: An examination of the theory of planned behaviour in the exercise domain. *British Journal of Social Psychology, 42*(1), 129-146. https://doi.org/10.1348/014466603763276162
11. Sallis, J. F., Grossman, R. M., Pinski, R. B., Patterson, T. L., & Nader, P. R. (1987). The development of scales to measure social support for diet and exercise behaviors. *Preventive Medicine, 16*(6), 825-836. https://doi.org/https://doi.org/10.1016/0091-7435(87)90022-3
12. Conner, M., Norman, P., & Bell, R. (2002). The theory of planned behavior and healthy eating. *Health Psychology, 21*(2), 194-201. https://doi.org/10.1037/0278-6133.21.2.194
13. Echeverria, S. E., Diez-Roux, A. V., & Link, B. G. (2004). Reliability of self-reported neighborhood characteristics. *Journal of Urban Health, 81*(4), 682-701. https://doi.org/10.1093/jurban/jth151
14. Dewar, D. L., Lubans, D. R., Plotnikoff, R. C., & Morgan, P. J. (2012). Development and evaluation of social cognitive measures related to adolescent dietary behaviors. *International Journal of Behavioral Nutrition and Physical Activity, 9*(1), 36-36. https://doi.org/10.1186/1479-5868-9-36
15. Anderson, D. F., & Cychosz, C. M. (1994). Development of an exercise identity scale. *Perceptual and Motor Skills, 78*(3), 747-751. https://doi.org/10.1177/003151259407800313
16. Carfora, V., Caso, D., & Conner, M. (2016). The role of self-identity in predicting fruit and vegetable intake. *Appetite, 106*, 23-29. https://doi.org/10.1016/j.appet.2015.12.020
17. Markland, D., & Ingledew, D. K. (1997). The measurement of exercise motives: Factorial validity and invariance across gender of a revised exercise motivations inventory. *British Journal of Health Psychology, 2*(4), 361-376. https://doi.org/10.1111/j.2044-8287.1997.tb00549.x
18. Turner-McGrievy, G. M., Wright, J. A., Migneault, J. P., Quintiliani, L., & Friedman, R. H. (2014). The interaction between dietary and life goals: Using goal systems theory to explore healthy diet and life goals. *Health Psychology and Behavioral Medicine, 2*(1), 759-769. https://doi.org/10.1080/21642850.2014.927737
19. Marcus, B. H., Rossi, J. S., Selby, V. C., Niaura, R. S., & Abrams, D. B. (1992). The stages and processes of exercise adoption and maintenance in a worksite sample. *Health Psychology, 11*(6), 386-395. https://doi.org/10.1037/0278-6133.11.6.386
20. Epel, E. S., Tomiyama, A. J., Mason, A. E., Laraia, B. A., Hartman, W., Ready, K., Acree, M., Adam, T. C., St Jeor, S., & Kessler, D. (2014). The reward-based eating drive scale: A self-report index of reward-based eating. *PLoS ONE, 9*(6), e101350. https://doi.org/10.1371/journal.pone.0101350
21. Thompson, E. R. (2007). Development and validation of an internationally reliable short-form of the positive and negative affect schedule (PANAS). *Journal of Cross-Cultural Psychology, 38*(2), 227-242. https://doi.org/10.1177/0022022106297301
22. Macht, M., & Simons, G. (2000). Emotions and eating in everyday life. *Appetite, 35*(1), 65-71. https://doi.org/10.1006/appe.2000.0325
