# Supplementary material for: Capability, opportunity, and motivation: an across contexts empirical examination of the COM-B model
Source: BMC Public Health. 2021 May 29;21:1014. doi: 10.1186/s12889-021-11019-w (PMC8164288; doi:10.1186/s12889-021-11019-w)
Supplement: Supplementary file 2 — Additional file 2. Survey instruments. Supplementary tables comprising English-language copies of the survey instruments (questionnaires) used in this study. [file 12889_2021_11019_MOESM2_ESM.pdf]

Additional File 2

Survey instruments

Physical activity survey instrument			
**Measure**	**No.**	**Response format**	**Item wording**
**Knowledge**	6	Nominal 'true or false' scale	Please indicate whether the following statements are true or false.
			Adults aged 18 - 64 years should engage in at least 150 minutes of moderate intensity (i.e., exercises requiring some effort, but conversation is still possible) physical activity each week.
			Adults aged 18-64 years should engage in at least 75 minutes of vigorous intensity (i.e., exercises which induce heaving breathing) physical activity each week.
			An equivalent combination of both moderate and vigorous activities each week is sufficient (e.g., 1.5 hrs of moderate intensity + 1 hr of vigorous intensity exercise) for adults aged 18-64 years.
			Adults aged 18-64 years should do muscle strengthening activities on at least 2 days each week.
			56% of Australian adults aged 18 years and over are either inactive or have low levels of physical activity.
			Regular physical activity can reduce your risk of cardiovascular disease, type 2 diabetes, and some cancers, but only if it is performed at a moderate-vigorous intensity.
**Decision Making**	16	7-point Likert scale ranging from strongly disagree to strongly agree	Please read the following statements and indicate your level of agreement when it comes to making the decision of whether to exercise or not.
			I would have more energy for my family and friends if I exercised regularly.
			Regular exercise would help me relieve tension.
			I would feel more confident if I exercised regularly.
			I would sleep more soundly if I exercised regularly.
			I would feel good about myself if I kept my commitment to exercise regularly.
			I would like my body better if I exercised regularly.
			It would be easier for me to perform routine physical tasks if I exercised regularly.
			I will feel less stressed if I exercise regularly.
			I would be more comfortable with my body if I exercised regularly.
			Regular exercise would help me have a more positive outlook on life.
			I think I would be too tired to do my daily work after exercising.
			I would find it difficult to find an exercise activity that I enjoy that is not affected by bad weather.
			I feel uncomfortable when I exercise because I get out of breath and my heart beats very fast.

**Physical activity survey instrument**			
**Measure**	**No.**	**Response format**	**Item wording**
			Regular exercise would take too much of my time.
			I would have less time for my family and friends if I exercised regularly.
			At the end of the day, I am too exhausted to exercise.
**Action Control**	6	7-point Likert scale ranging from very untrue to very true	Think about the last four weeks and indicate your level of agreement with the following statements. During the last four weeks I have...
			Constantly monitored whether I exercise frequently enough.
			Watched carefully that I exercise for at least 30 minutes at a moderate-intense level.
			I have had my exercise intention often on my mind.
			I have always been aware of my planned exercise regime.
			I have really tried to exercise regularly.
			Tried my best to act in accordance with my standards (for exercise).
**Action Planning**	4	7-point Likert scale ranging from very untrue to very true	Think about the last four weeks and indicate your level of agreement with the following statements. During the last four weeks I have made a detail planned regarding...
			When to do my physical exercise.
			Where to exercise.
			How to do my physical exercise.
			How often to do my physical exercise.
**Habit**	12	7-point Likert scale ranging from strongly disagree to strongly agree	Please indicate your level of agreement with the following statements. Physical activity is something...
			I do frequently.
			I do automatically.
			I do without having to consciously remember.
			That makes me feel weird if I do not do it.
			I do without thinking.
			That would require effort not to do it.
			That belongs to my (daily, weekly, monthly) routine.
			I start doing before I realise, I’m doing it.
			I would find hard not to do.
			I have no need to think about doing.

**Physical activity survey instrument**			
**Measure**	**No.**	**Response format**	**Item wording**
			That's typically "me."
			I have been doing for a long time.
**Skills/Competence**	6	7-point Likert scale ranging from very untrue to very true	Please indicate which of the following statements most describes you.
			I do very well at all kinds of physical activities. [Reverse coded]
			I wish I could be a lot better at physical activities.
			I think I could do well at just about any new physical activity I haven't tried before. [Reverse coded]
			In physical activities I usually watch instead of participating.
			I feel that others my age are generally better than me at physical activities.
			I don't feel I can do well at new physical activities.
**Environment**	11	5-point Likert scale ranging from strongly disagree to strongly agree	Please read the following statements and indicate your level of agreement.
			My neighbourhood offers many opportunities to be physically active.
			Local sports clubs and other similar providers in my neighbourhood offer many opportunities to get exercise.
			It is pleasant to walk in my neighbourhood.
			There are enough trees in my neighbourhood to provide shade.
			My neighbourhood has heavy traffic. [Reverse coded]
			There are busy roads to cross when out for walks in my neighbourhood. [Reverse coded]
			In my neighbourhood it is easy to walk to places.
			There are stores within walking distance of my home.
			In my neighbourhood, the streets and sidewalk are in good condition.
			I often see other people walking in my neighbourhood.
			I often see other people exercising (e.g., jogging, cycling, playing sports) in my neighbourhood.
**Resources**	8	Nominal 'yes or no' scale	Please indicate if there are any of the following facilities within a 20-minute walk from your neighbourhood.
			Public park.
			Public (or private) sports field, basketball court, or tennis court.
			Public (or private) pool or beach.

Physical activity survey instrument			
**Measure**	**No.**	**Response format**	**Item wording**
			Schools, colleges, or community centres with recreational facilities that are free and open to the public.
			Gyms, health/fitness clubs, or pools that you must join and pay for.
			PCYC, YMCA, YWCAs, or similar facilities.
			Bicycle path in the street or park.
			Sidewalks.
**Subjective Norms**	5	7-point Likert scale ranging from strongly disagree to strongly agree	Please read the following statements and indicate your level of agreement.
			Most people in my social network want me to exercise regularly in the next 2 weeks.
			Most people in my social network would approve if I exercised regularly in the next 2 weeks.
			Most of my friends exercise regularly,
			Most of my family members exercise regularly.
			Most of my co-workers exercise regularly.
**Social Support**	13	7-point Likert scale ranging from never to always	During the past 12 months, how often have your family, friends, and experts...
			Exercised with me.
			Offered to exercise with me.
			Gave me helpful reminders to exercise (“Are you going to exercise tonight?”).
			Gave me encouragement to stick with my exercise program.
			Changed their schedule so we could exercise together.
			Discussed exercise with me.
			Complained about the time I spend exercising. [Reverse coded]
			Criticised me or made fun of me for exercising. [Reverse coded]
			Gave me rewards for exercising (bought me something or gave me something I like).
			Planned for exercise on recreational outings
			Helped plan activities around my exercise.
			Asked me for ideas on how they can get more exercise.
			Talked about how much they like to exercise.
**Self-Efficacy**	3	7-point Likert scales*	Please read the following statements and indicate your level of confidence or agreement.

Physical activity survey instrument			
**Measure**	**No.**	**Response format**	**Item wording**
		*Very unconfident to very confident	How confident are you that you will be able to exercise regularly in the next 2 weeks?
		*Very unconfident to very confident	How confident are you over the next 2 weeks that you could overcome obstacles that prevent you from exercising regularly?
		*Very untrue to very true	I believe I have the ability to regularly exercise in the next 2 weeks.
**PBC**	3	7-point Likert scales*	Please read the following statement and indicate the level of agreement or control you feel.
		*Strongly disagree to strongly agree	Whether or not I exercise regularly in the next 2 weeks is entirely up to me.
		*No control to complete control	How much personal control do you feel you have over exercising regularly in the next 2 weeks?
		*Completely beyond my control to very much within my control	How much do you feel that exercising regularly in the next 2 weeks is within your control?
**Attitudes**	6	7-point semantic differential scale	For me, exercising regularly over the next 2 weeks would be...
			Harmful | Beneficial
			Useless | Useful
			Foolish | Wise
			Boring | Interesting
			Unenjoyable | Enjoyable
			Stressful | Relaxing
**Goals**	12	7-point Likert scale ranging from very untrue to very true	Personally, I exercise (or might exercise) …
			To spend time with friends.
			To enjoy the social aspects of exercising.
			To have fun being active with other people.
			To make new friends.
			To help me look younger.
			To have a good body.
			To improve my appearance.
			To look more attractive.
			To give me goals to work towards.
			To give me personal challenges to face.
			To develop personal skills.

Physical activity survey instrument			
**Measure**	**No.**	**Response format**	**Item wording**
			To measure myself against personal standards.
**Intentions**	3	7-point Likert scale ranging from strongly disagree to strongly agree	Over the next three months I...
			Intend to engage in at least 30 minutes of moderate intensity physical activity, five times per week.
			Determined to engage in at least 30 minutes of moderate intensity physical activity, five times per week.
			Plan to engage in at least 30 minutes of moderate intensity physical activity, five times per week.
**Identity**	9	7-point Likert scale ranging from strongly disagree to strongly agree	The following questions concern your personal beliefs about exercise. Please indicate the degree to which you agree or disagree with each statement when thinking about your exercise participation.
			I consider myself an exerciser.
			When I describe myself to others, I usually include my involvement in exercise.
			I have numerous goals related to exercising.
			Physical exercise is a central factor to my self-concept.
			I need to exercise to feel good about myself.
			Others see me as someone who exercises regularly.
			For me, being an exerciser means more than just exercising.
			I would feel a real loss if I were forced to give up exercising.
			Exercising is something I think about often.
**Affect/Emotions**	10	5-point Likert scale ranging from never to always	Thinking about yourself and how you normally feel, to what extent do you generally feel the following emotions about physical activity.
			Determined.
			Attentive.
			Alert.
			Inspired.
			Active.
			Afraid.
			Nervous.
			Upset.
			Ashamed.

Physical activity survey instrument			
**Measure**	**No.**	**Response format**	**Item wording**
			Hostile.
**Reinforcement**	4	5-point Likert scale ranging from never to always	Please indicate how often you do the following after exercising.
			I reward myself when I exercise.
			I try to set realistic goals for myself rather than setting myself up for failure by expecting too much.
			When I exercise, I tell myself that I am being good to myself by taking care of my body in this way.
			I do something nice for myself for making efforts to exercise more.
**Behaviour (IPAQ-SF)**	7	Ratio and nominal scales	
		Days per week	During the last 7 days, on how many days did you do **vigorous** physical activities like heavy lifting, digging, aerobics, or fast bicycling?
		Hours and minutes per day	How much time did you usually spend doing vigorous physical activities on one of those days?
		Days per week	During the last 7 days, on how many days did you do **moderate** physical activities like carrying light loads, bicycling at a regular pace, or doubles tennis? Does not include walking.
		Hours and minutes per day	How much time did you usually spend doing moderate physical activities on one of those days?
		Days per week	During the last 7 days, on how many days did you walk for at least 10 minutes at a time?
		Hours and minutes per day	How much did you usually spend walking on one of those days?
		Hours and minutes per day	During the last 7 days, how much time did you spend sitting on a weekday?

Note. See Additional File 1 for measure sources. Abbreviations: IPAQ = International Physical Activity Questionnaire – Short Form; PBC = perceived behavioural control.

Eating survey instrument			
**Measure**	**No.**	**Response format**	**Item wording**
**Knowledge**	8	Nominal 'true or false' scale	Please indicate whether you believe the following statements are true or false.
			A balanced diet implies eating all foods in the same amounts.
			The health benefit of fruit and vegetables lies alone in the supply of vitamins and minerals.
			The same amount of beef steak and chicken breast contains equally as many calories.
			The same amount of sugar and fat contains equally as many calories.
			Fat is always bad for your health.
			To eat healthily, you should eat less fat.
			A healthy meal should consist of half meat, a quarter vegetable, and a quarter side dishes.
			For a healthy nutrition, dairy products should be consumed in the same amounts as fruit and vegetables.
**Decision Making**	15	7-point Likert scale ranging from strongly disagree to strongly agree	Please read the following statements carefully and indicate your level of agreement with each.
			I would have more energy if I ate healthily.
			I would be doing something good for my body if I ate healthily.
			I would 'feel' good if I ate healthily.
			Eating healthily would help me manage my weight.
			I enjoy the taste of healthy foods.
			It takes too much time to prepare healthy foods.
			I would rather eat sweets or high fat snacks than healthy foods.
			Healthy foods do not satisfy my hunger for very long.
			Healthy foods are too expensive.
			I don't like the taste of healthy foods.
**Action Control**	6	7-point Likert scale ranging from strongly disagree to strongly agree	Think about the last four weeks and indicate your level of agreement with the following statements. During the last four weeks I have...
			Constantly monitored whether I am eating a healthy diet.
			Watched carefully that I eat the recommended 2 servings of fruit and 5 servings of vegetables each day.
			Had my healthy eating intention on my mind.

Eating survey instrument			
**Measure**	**No.**	**Response format**	**Item wording**
			Always been aware of my plan to eat a healthy diet.
			Really tried to eat healthily on most days of the week.
			Tried my best to act in accordance with my standards (in relation to eating healthily).
**Action Planning**	4	7-point Likert scale ranging from very untrue to very true	Think about the last four weeks and indicate the level to which the following statements are untrue/true for you. During the last four weeks I have made a detailed plan regarding...
			When to do my healthy meal preparations for the week.
			Where to shop for my healthy meal preparations.
			How I will approach my meal preparation for the week (e.g., cookbook, download recipes from online website, download an app etc.).
			How often to do my meal preparations (e.g., cook meals for next 2-3 days only OR cook and freeze pre-made meals for the entire week).
**Habit**	12	7-point Likert scale ranging from strongly disagree to strongly agree	Please indicate your level of agreement with the following statements. Healthy eating is something...
			I do frequently.
			I do automatically.
			I do without having to consciously remember.
			that makes me feel guilty if I do not do it.
			I do without thinking.
			I find effortless to do (e.g., not eating healthy would require more effort than eating healthy for you).
			that belongs to my (daily, weekly, monthly) routine (e.g., you eat a healthy diet every day).
			that requires little thought.
			I would find hard not to do.
			I have no need to think about doing.
			That’s typically “me.”
			I have been doing for a long time.
**Skills/Competence**	7	7-point Likert scale ranging from strongly disagree to strongly agree	Please indicate which of the following statements most describes you.

**Eating survey instrument**			
**Measure**	**No.**	**Response format**	**Item wording**
			I can cook complicated multi-course meals.
			I can prepare a lot of meals even without a recipe.
			I can prepare gratin (basic culinary technique - potato topped with a browned crust).
			I can prepare a soup.
			I can prepare a sauce.
			I can bake a cake.
			I can bake bread.
**Environment**	10	7-point Likert scale ranging from strongly disagree to strongly agree	Please indicate your level of agreement with the following statements.
			At work there is a wide variety of healthy food options to choose from.
			At work there is a wide variety of healthy drink options to choose from.
			At work there is at least one option where I have healthy selections to choose from.
			There is a wide variety of fresh fruits and vegetables where I shop.
			The fruits and vegetables where I shop are at good prices.
			The fruits and vegetables where I shop are of good quality.
			At home there are healthy snacks available to eat.
			At home there are healthy drinks available.
			At home fruit is always available to eat.
			At home vegetables are always available to eat.
**Subjective Norms**	6	7-point Likert scale ranging from strongly disagree to strongly agree	Please read the following statements carefully and indicate your level of agreement.
			Most people in my social network think I should eat a healthy diet in the next 2 weeks.
			Most people in my social network want me to eat a healthy diet in the next 2 weeks.
			Most people in my social network would approve if I ate a healthy diet in the next 2 weeks.
			Most of my friends eat a healthy diet.
			Most of my family eat a healthy diet.
			Most of my co-workers eat a healthy diet.

Eating survey instrument			
**Measure**	**No.**	**Response format**	**Item wording**
**Social Support**	10	5-point Likert scale ranging from almost never to almost always	How often in the past month (30 days) has your family or friends done the following?
			Encouraged you to eat healthy foods.
			Discussed the benefits of eating healthy foods with you.
			Reminded you to eat healthy foods.
			Complimented you on your healthy eating habits.
			Commented if you went back to old “unhealthy” eating habits.
			Ate unhealthy foods in front of you.
			Refused to eat healthy foods with you.
			Brought home foods you were trying not to eat.
			Complained about eating healthy foods.
			Offered me food I’m trying not to eat.
**Self-Efficacy**	3	7-point Likert scales*	Please read the following statements and indicate your level of confidence, difficulty, or perceived ability.
		*Very unconfident to very confident	How confident are you that you will be able to eat healthily over the next 2 weeks?
		*Very difficult to very easy	For me to eat healthily over the next 2 weeks would be:
		*Very untrue to very true	I believe I have the ability to eat healthily in the next 2 weeks.
**PBC**	3	7-point Likert scales*	Please read the following statement and indicate your level of agreement.
		*Strongly disagree to strongly agree	Whether or not I eat healthily in the next 2 weeks is entirely up to me.
		*No control to complete control	How much control do you feel you have overeating healthily in the next 2 weeks?
		*Completely beyond my control to very much within my control	How much do you feel that eating healthily in the next 2 weeks is within your control?
**Attitudes**	6	7-point semantic differential scale	For me, eating a healthy diet over the next 2 weeks would be...
			Good | Bad
			Harmful | Beneficial
			Foolish | Wise
			Unenjoyable | Enjoyable
			Unpleasant | Pleasant
			Stressful | Relaxing

Eating survey instrument			
**Measure**	**No.**	**Response format**	**Item wording**
**Goals**	10	7-point Likert scale ranging from very untrue to very true	In the past month, how often have you set a goal to...
			Eat more fruits.
			Eat more vegetables.
			Eat less sweets.
			Eat fewer fast food/take-out.
			Drink more water.
			Drink less sugar-sweetened drinks.
			Drink less caffeine.
			Drink less alcohol (e.g., beer, wine, hard liquor).
			Eat a balanced diet.
			Maintain healthy changes that you have already made to your diet.
**Intentions**	5	7-point Likert scale ranging from strongly disagree to strongly agree	Over the next three months I...
			Intend to eat a healthy diet.
			Will try to eat a healthy diet.
			Want to eat a healthy diet.
			Expect to eat a healthy diet.
			How likely is it that you will eat a healthy diet over the next three months?
**Identity**	9	7-point Likert scale ranging from strongly disagree to strongly agree	Please indicate your level of agreement with the following statements.
			I think of myself as a healthy eater.
			I think of myself as a person who is interested in eating healthy.
			I think of myself as someone who is concerned about the health consequences of what I eat.
**Affect/Emotions**	10	5-point Likert scale ranging from not at all associated to very strongly associated	Please indicate the extent to which you associate the following emotions with eating.
			Feeling of anger (e.g., angry/annoyed).
			Feeling of fear (e.g., anxious/fearful).
			Feeling of sadness (e.g., sad/depressed).

Eating survey instrument			
**Measure**	**No.**	**Response format**	**Item wording**
			Feeling of joy (e.g., joyful/happy).
			Feeling of tension (e.g., tense/excited).
			Feeling of relaxation (e.g., at ease/relaxed).
**Motivation**	10	7-point Likert scale ranging from very untrue to very true	Please indicate the extent to which the following statements are true for you.
			I eat when I have feelings of hunger.
			I eat when I have a desire to eat.
			I eat when I have bodily symptoms of hunger (e.g., empty feeling in stomach, stomach rumbling).
			I have a tendency to eat irregularly.
			I have a tendency to eat sweet food (e.g., chocolate).
			I have a tendency to eat good tasting food.
			I have a tendency to enjoy food.
			I have a tendency to eat to provide distraction.
			I have a tendency to eat something to relax.
			I have a tendency to eat to feel better.
**Reinforcement**	9	7-point Likert scale ranging from strongly disagree to strongly agree	Please indicate how often you do the following after exercising.
			I feel out of control in the presence of delicious food.
			When I start eating, I just can't seem to stop.
			It is difficult for me to leave food on my plate.
			When it comes to foods I love, I have no willpower.
			I get so hungry that my stomach often seems like a bottomless pit.
			I don't get full easily.
			It seems like most of my waking hours are preoccupied by thoughts about eating or not eating.
			I have days when I can't seem to think about anything else but food.
			Food is always on my mind.
**Behaviour**	12	Nominal, interval, and ratio scales.	

		**Eating survey instrument**	
**Measure**	**No.**	**Response format**	**Item wording**
		11-point scale ranging from never to > 10 serves	How many serves of vegetables do you eat on a usual day?
		11-point scale ranging from never to > 10 serves	How many serves of fruit do you eat on a usual day?
		12-point scale ranging from never to > 10 times per week	On average, how many times per week do you drink soft drinks (including flavoured or mineral water)?
		12-point scale ranging from never to > 10 times per week	In an average week, how many times would you eat something from a fast-food restaurant like McDonald's, Hungry Jacks, KFC, etc.
		11-point scale ranging from never to three or more times day	How often do you eat biscuits, cakes, chocolate, lollies, ice cream or crisps?
		I don’t eat bread<br>High fibre white bread<br>White bread<br>Wholemeal bread<br>Rye bread<br>Wholegrain or multi-grain bread<br>Gluten/wheat free bread	What type of bread do you usually eat?
		I do not eat rice, pasta or noodles<br>White<br>Brown/wholemeal/wholegrain<br>Gluten/wheat free	What type of rice, pasta or noodles do you mainly choose?
		I do not consume milk<br>Full cream milk (including lactose free)<br>Reduced fat milk (including lactose free)<br>Skim milk (including lactose free)<br>Soy/rice milk	What type of milk do you mainly choose?
		I do not eat fish<br>White (cod, hoki, whiting, etc.)<br>Oily (salmon, sardines, tuna, mackerel, trevally)	What types of fish do you usually choose to eat?
		12-point scale ranging from never to > 10 times per week	On average, how many times per week do you eat red meat?
		12-point scale ranging from never to > 10 times per week	On average, how many times per week do you eat meat products (such as sausages, frankfurter, Devon, fritz, salami, meat pies, bacon, or ham)?
		5-point Likert scale ranging from never to always	How often do you add salt to your food during or after it is cooked?

Note. See Additional File 1 for measure sources. Abbreviations: PBC = perceived behavioural control.

Sample characteristic questions	
**Sociodemographic**	Items
	What is your age (in years)?
	What is your sex?
	What is your highest level of education?
	What is your current employment status?
	What is your total annual household income (before tax)?
	What is current relationship status?
	Please indicate your ethnicity (i.e., your feeling of belonging and attachment to a distinct group of a larger population that shares your ancestry, colour, language, or religion) from the options provided below.
	Please provide your residential postcode (e.g., 4001).
**BMI**	**Items**
	What is your height in centimetres (cm)?
	What is your current weight in kilograms (kg)?

Note. Same items used for both surveys. Abbreviations: BMI = body mass index.
