# Supplementary material for: Capability, opportunity, and motivation: an across contexts empirical examination of the COM-B model
Source: BMC Public Health. 2021 May 29;21:1014. doi: 10.1186/s12889-021-11019-w (PMC8164288; doi:10.1186/s12889-021-11019-w)
Supplement: Supplementary file 3 — Additional file 3. Sample characteristics for both surveys (eating and physical activity). Supplementary table summarising key sample characteristics across datasets (eating and physical activity). [file 12889_2021_11019_MOESM3_ESM.pdf]

Additional File 3

Sample characteristics (eating and physical activity surveys)

	Eating		Physical Activity		Sig.
Socio-demographic	*N*	Mean (SD) or %	*N*	Mean (SD) or %	
Age	455	24.91 (5.15)	582	22.81 (4.87)	.000
BMI	455	24.21 (5.65)	582	20.30 (4.60)	.000
Diet score	455	78.03 (15.13)			
Total PA (mins/wk.)			582	152.48 (91.9)	
Total MVPA (mins/wk.)			582	90.79 (71.46)	
Sex					.744
Female	367	80.8	467	80.3	
Male	85	18.7	115	19.7	
Prefer not to answer	2	0.4	-	-	
Education					.000
Postgraduate degree	97	21.3	47	8.1	
Undergraduate degree	156	34.4	216	37.1	
Vocational qualification	69	15.2	82	14.1	
Year 12 or equivalent	131	28.9	234	40.2	
Less than year 12	1	0.2	3	0.05	
Employment					.087
Full-time work	97	21.4	53	9.1	
Part-time work	69	15.2	134	23.1	
Full-time student	237	52.2	336	57.7	
Part-time student	22	4.8		-	
Unemployed	21	4.6	59	10.2	
Other	8	1.8			
Annual household income (before tax)					.000
$0 - $25,000	129	28.7	262	45.1	
$25,001 - $50,000	78	17.3	133	22.8	
$50,001 - $75,000	60	13.3	58.2	10.0	
$75,001 - $100,000	38	8.4	51	8.8	
$100,001 - $150,000	49	10.9	54	9.3	
More than $150,001	31	6.9	23	4.0	
Prefer not to answer	65	14.4	-	-	
Relationship status					.085
Married	74	16.4	69	11.9	
Living with partner	108	23.9	129	22.2	
Single	225	49.8	384	65.9	
Other	45	10.0	-		
Ethnicity					.000
Caucasian	329	72.5	480	82.5	
Latino/Hispanic	5	1.1	4	0.6	
Middle Eastern	3	0.7	4	0.7	
South Asian	30	6.6	28	4.8	
East Asian	29	6.4	25	4.3	
Mixed	36	7.9	34	5.8	
Other	22	4.8	8	1.3	

Note. Age range = 18-35 years. Independent samples t-tests were conducted to ascertain between sample differences. Abbreviations: BMI = body mass index; MVPA = moderate to vigorous physical activity; PA = physical activity.
