# Supplementary material for: Capability, opportunity, and motivation: an across contexts empirical examination of the COM-B model
Source: BMC Public Health. 2021 May 29;21:1014. doi: 10.1186/s12889-021-11019-w (PMC8164288; doi:10.1186/s12889-021-11019-w)
Supplement: Supplementary file 4 — Additional file 4. Physical activity measurement model. Diagrammatic summary of physical activity measurement model. [file 12889_2021_11019_MOESM4_ESM.pdf]

Additional File 4

Physical activity measurement model

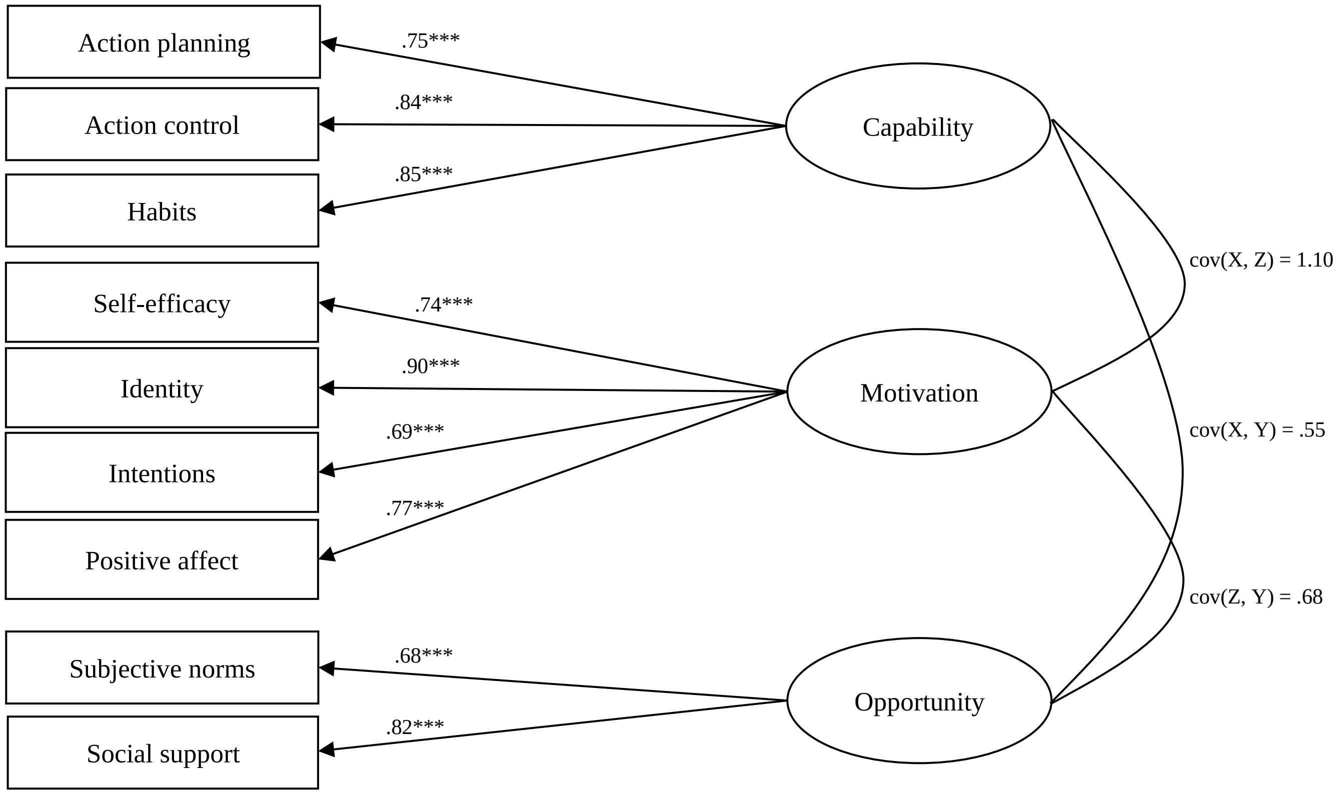

Note. Trimmed measurement model (N = 582). Model fit: CMIN/DF = 1.361; GFI = .989, AGFI = .977; CFI = .998; TLI = .996; RMSEA = .025; SRMR = .014.
Significance levels: *p < .05, **p < .01, ***p < .001.
