# Supplementary material for: Capability, opportunity, and motivation: an across contexts empirical examination of the COM-B model
Source: BMC Public Health. 2021 May 29;21:1014. doi: 10.1186/s12889-021-11019-w (PMC8164288; doi:10.1186/s12889-021-11019-w)
Supplement: Supplementary file 5 — Additional file 5. Eating measurement model. Diagrammatic summary of eating measurement model. [file 12889_2021_11019_MOESM5_ESM.pdf]

Additional File 5

Eating measurement model

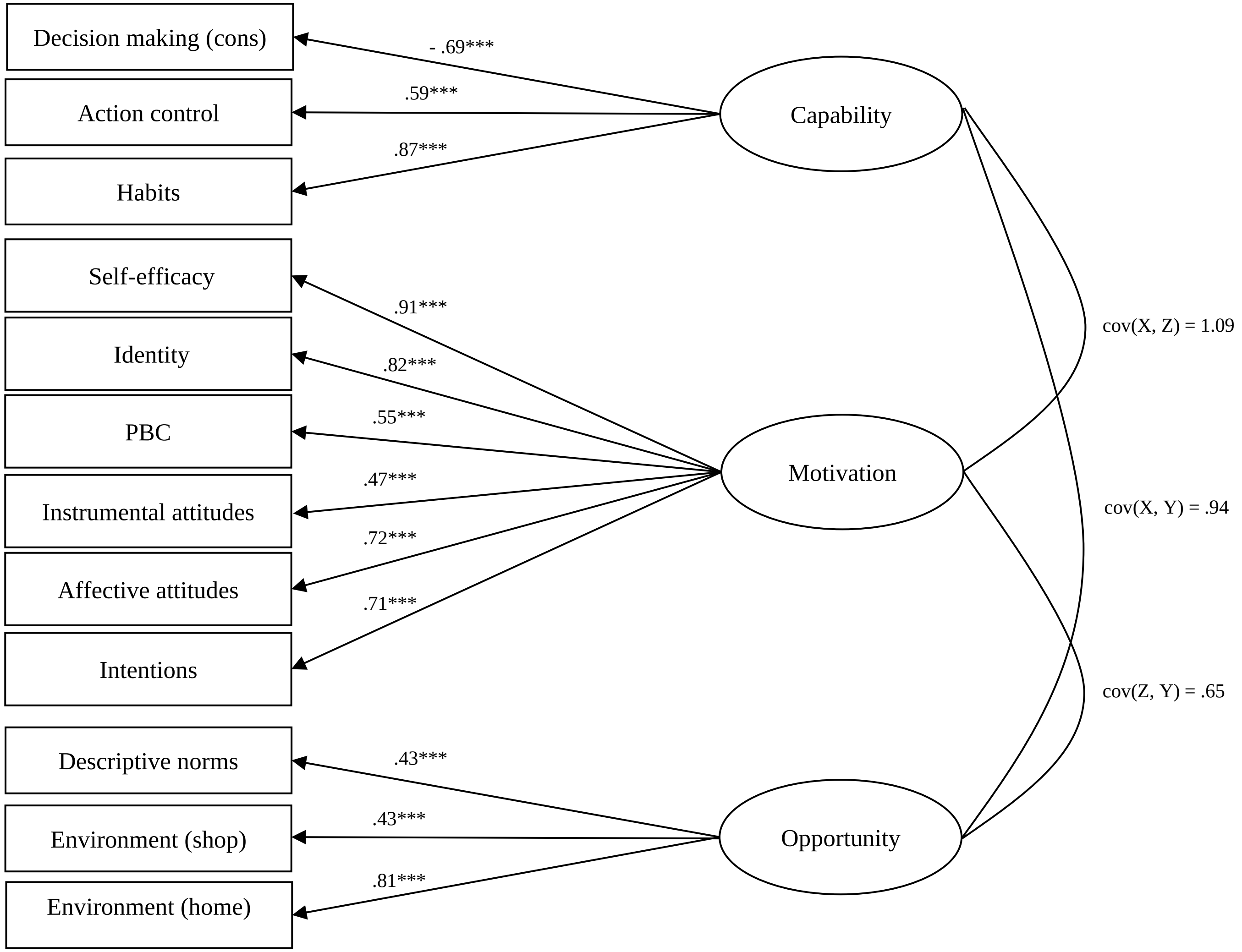

Note. Trimmed model (N = 455). Model fit: CMIN/DF = 1.957; GFI = .974, AGFI = .945; CFI = .987; TLI = .976; RMSEA = .046; SRMR = .026.
Significance levels: *p < .05, **p < .01, ***p < .001.
